# Fatal Postpartum Hemorrhage in a Patient with Niemann-Pick Disease Type B

**DOI:** 10.1155/2018/9719374

**Published:** 2018-06-28

**Authors:** Atakan Tanacan, Abdullah Yalcin, Canan Unal, Seda Banu Akinci, Mehmet Sinan Beksac

**Affiliations:** ^1^Division of Perinatology, Department of Obstetrics and Gynecology, Hacettepe University Medical Faculty, Ankara, Turkey; ^2^Department of Anaesthesiology and Critical Care Medicine, Hacettepe University Medical Faculty, Ankara, Turkey

## Abstract

**Background:**

Niemann-Pick Disease Type B (NPD B) is a rare lysosomal storage disorder resulting from an inherited deficiency of acid sphingomyelinase activity. Here, we report the case of a splenectomized patient with NPD B who died because of severe postpartum hemorrhage (PPH).

**Case Presentation:**

A 23-year-old nulliparous woman was admitted to intensive care unit (ICU) after cardiopulmonary arrest during urgent hysterectomy because of severe postpartum bleeding. The patient concealed her disease from her family and obstetricians during her pregnancy, and her NPD B diagnosis was revealed during her stay in ICU while searching for the cause of the splenectomy and severe bleeding. Unfortunately, she had a detrimental course with hypoxic brain injury leading to brain death.

**Conclusions:**

In conclusion, physicians should keep in mind that patients with a history of splenectomy and/or uncontrollable hemorrhage must be carefully evaluated for rare diseases like lysosomal storage diseases and that NPD B can cause mortality because of postpartum bleeding. Adult intensivists should be familiar with adult presentations of rare metabolic or genetic diseases as more and more children with metabolic or genetic diseases will survive to adulthood and will be admitted to and unfortunately will even die in the adult ICU.

## 1. Introduction

Niemann-Pick Disease Types A and B (NPD A and B) are lysosomal storage disorders resulting from an inherited deficiency of acid sphingomyelinase (ASM) activity [1, 2]. Both types are inherited as autosomal recessive traits, which are caused by homozygous or compound heterozygous mutations in the sphingomyelin phosphodiesterase-1 gene (SMPD1) on chromosome 11p15 [3]. It has an estimated birth prevalence of 0.4-0.6/100.000 [4].

Deficient ASM activity leads to a progressive accumulation of sphingomyelin, cholesterol, and other lipids within the reticuloendothelial cells, visceral organs, and neurons of the affected patients [2]. NPD A, which is the infantile form of the disease, causes severe neurodegeneration and results in death by 3 years of age. However, patients with NPD B mostly survive into adulthood, depending on the severity of the disease. Neurological involvement is rare, and visceral organs (especially in the spleen, liver, and lungs) are typically affected in NPD B. Thrombocytopenia, anemia, atherosclerosis, and osteopenia/osteoporosis are the other serious complications related to NPD B [3]. Clinical findings of NPD B may include hepatosplenomegaly, hyperlipidemia, infiltrative pulmonary disease, liver dysfunction, cardiac disease, retinal stigmata, growth retardation, skeletal deformities, and neurodegeneration in some rare cases [1, 3]. On the other hand, clinical features vary significantly among patients. While some patients may present with only mild organomegaly or slightly elevated liver enzymes, some others may present with serious complications like severe organomegaly, cirrhosis, pulmonary insufficiency, fatal bleeding, or coronary heart disease [1, 3]. Furthermore, the onset of the disease varies from early childhood to the fifth decade of life [5]. No specific treatment has been established for NPD A yet [6]. Conservative management protocols such as lowering serum cholesterol levels, supplementation of oxygen, and transfusion of blood products may be performed for NPD B [3]. Additionally, bone marrow transplantation resulted in reduction in spleen and liver volumes and increased peripheral blood cell counts, together with decreased infiltration of the lungs in some patients with NPD B [3, 6]. Although enzyme replacement therapy is not a routine part of the standard care protocol in NPD for now, researchers are working on early phase clinical trials in order to evaluate the efficacy of recombinant ASM for the treatment of the nonneurologic manifestations of adults with NPD B [7].

Postpartum hemorrhage (PPH) is the leading cause of maternal mortality worldwide, and it is classically defined as “the loss of 500 ml or more of blood from the genital tract after the delivery of the baby” by the World Health Organization (WHO) [8]. Severe PPH, which is defined as “genital bleeding after delivery, with at least one of the following: perceived abnormal bleeding (1000 ml or more) or any bleeding with hypotension or blood transfusion”, may lead to maternal mortality and morbidity, and it is an obstetric emergency condition that has to be managed meticulously by physicians [8].

Here, we present a case of maternal mortality due to fatal PPH in a patient with NPD B.

## 2. Case Presentation

A 23-year-old nulliparous woman was admitted to a state hospital in Ankara Hospital with regular uterine contractions at 40 weeks of her pregnancy. She had no known prenatal risk factor except a history of a splenectomy, which was performed because of trauma-related hemorrhage according to her statement. Cesarean section (CS) was performed for obstructed labor without any complication, and severe PPH was diagnosed sixteen hours after the surgery. A postpartum hysterectomy was performed urgently because of uncontrolled bleeding. Persistent tachycardia and hypotension were recorded during the surgery and prehysterectomy hemoglobin value of 4 mg/dl was reported. Six units of erythrocyte suspension and four units of fresh frozen plasma were given during the surgery for replacement of the lost blood. Unfortunately, cardiopulmonary arrest (CPA) developed in the last stages of the surgery, and cardiopulmonary resuscitation (CPR) was performed for 40 minutes until spontaneous heart beats began. The patient could not be extubated after the surgery and neurological examination revealed early signs of cerebral ischemia. Then, the patient was taken to Hacettepe University Hospital for intensive care and further evaluation.

She had fixed bilateral dilated pupils, her Glasgow Coma Scale (GCS) was three, her body temperature was 33 centigrade degrees, her blood pressure was 143/70 mmHg (MAP=97), her heart rate was 120 beats per minute, and arterial pH was 6.81. Extensive periphery edema was observed, and moist rales were auscultated, which indicated the onset of pulmonary edema. Pneumothorax in the apical lobe of right lung, interlobular septal thickening, and ARDS findings were detected in thorax CT. Mechanical ventilation was applied with positive pressure and positive end-expiratory pressure (PEEP). Complete blood count, blood biochemistry, arterial blood gas, coagulation profile, C-reactive protein (CRP), disseminated intravascular coagulation (DIC) panel, cardiac enzymes, electrocardiography (ECG), and posteroanterior chest X-ray were evaluated. Multiple organ failure due to hypovolemic shock was diagnosed. Central vascular access was established. Fluid replacement and inotropic support with noradrenaline and dopamine were applied. The patient began to have treatment-resistant tonic-clonic seizures; antiepileptic and anesthetic drugs were given to stop the seizures. Targeted temperature management was started with surface temperature-management device. Hypothermia (34°C) was applied for the first 24 hours. Then normothermia (36°C) was applied for the next three days. The seizures were stopped after three days. Broad spectrum antibiotics (meropenem and colistin) were administrated. Cranial magnetic resonance imaging (MRI) revealed severe hypoxic ischemic encephalopathy and bilateral cerebellar herniation.

During the search for the possible medical cause of the severe bleeding the patient's past medical record was evaluated, and it was found out that the patient had splenectomy at the age of sixteen. To our surprise, the consultation reports seven years ago revealed the diagnosis of NPD B. She received reconsultation from the pediatric metabolism and pediatric gastroenterology divisions which revealed that her parents had a consanguineous marriage, and she also had an older brother with NPD B.

There was no sign of gaining consciousness one week after cessation of the sedative drugs and no brain stem reflexes were obtained. Apnea test was positive and cerebral CT angiography was compatible with brain death. On the 14th day at the intensive care unit, brain death was confirmed. Brain death was declared to the patient's relatives but they did not donate the organs of the patient. Two days after the patient was pronounced as brain dead, the patient died after cardiac arrest.

## 3. Discussion

PPH accounts for 18% of maternal deaths in Turkey according to a recent study [9]. Despite the improvements in prenatal and postnatal care programs, PPH is still one of the leading causes of maternal mortality and morbidity in our country [9]. PPH can result from various obstetric reasons. Uterine atony, maternal bleeding disorders, abnormal placentation, placental retention, uterine inversion, and postpartum infection are some of the most frequent causes of PPH [10].

NPD A and NPD B are rare inherited lysosomal storage disorders [1, 2]. Patients with NPD B can survive into adulthood, and they can present with various complications depending on the disease severity. The primary causes of death in patients with NPD B were found to be respiratory failure and serious liver disease [1, 3]. Bleeding complications, heart failure, complications related to bone marrow transplant, neurodegeneration, and malignancies were the other reasons of death in these patients [1, 3]. Early onset of disease and undergoing a splenectomy are two main factors that increase the mortality rate, as these two factors give an indication of the severity of the disease, according to recent studies [1, 3]. Trauma-related bleeding, postoperative hemorrhage, splenic vein tear, and gastrointestinal bleeding/varices bleeding were the leading causes of death related to hemorrhage in patients with NPD B [1, 3]. Splenomegaly, thrombocytopenia, bone marrow involvement, and end-stage liver disease seemed to be the main reasons behind the bleeding disorders in these patients. However, to the best of our knowledge, there was no reported case in the literature of a patient who died of PPH. Another important point was that the patient concealed her disease from her family and obstetricians most probably because of personal reasons.

In conclusion, physicians should keep in mind that patients with a history of splenectomy and/or uncontrollable hemorrhage must be carefully evaluated for rare diseases like lysosomal storage diseases. Adult intensivists should be familiar with adult presentations of rare metabolic or genetic diseases as more and more children with metabolic or genetic diseases will survive to adulthood and will be admitted to and unfortunately will even die in the adult ICU. Lastly, we can also conclude that NPD B can lead to mortality because of postoperative bleeding.

## Data Availability

The datasets used and/or analysed during the current study are available from the corresponding author on reasonable request.
